# BCL6 regulates skeletal muscle mass and mitochondrial bioenergetics

**DOI:** 10.1016/j.molmet.2026.102367

**Published:** 2026-04-13

**Authors:** Shirine E. Usmani, Jean-Philippe Leduc-Gaudet, Stephanie Chau, Catherine A. Bellissimo, Shabana Vohra, Krystel Desjardins, Evan Pollock-Tahiri, Irisa Shi, Felice Chun, Anthony Capobianco, Pascale Delisle, Wanda Dupebe, Marina Cefis, Vincent Marcangeli, Martha Ghebreselassie, Yu-Cheng Liang, Yasufumi Seki, Dominique Mayaki, Sabah Hussain, Ewan Goligher, Mohit Kapoor, Marius Locke, Gilles Gouspillou, Minna Woo

**Affiliations:** 1 University Health Network Research Institute, University Health Network, Toronto, ON, Canada; 2 Division of Endocrinology & Metabolism, Department of Medicine, University Health Network, Toronto, ON, Canada; 3 Research Group in Cellular Signaling, Department of Medical Biology, Université du Québec À Trois-Rivières, Trois-Rivières, QC, Canada; 4 Department of Pharmacology & Toxicology, University of Toronto, Toronto, ON, Canada; 5 Département des sciences de l'activité physique, Faculté des sciences, Université du Québec à Montréal, Montréal, QC, Canada; 6 Schroeder Arthritis Institute, University Health Network, Toronto, ON, Canada; 7 INSERM UMR1093-CAPS, Université de Bourgogne, UFR des Sciences de Santé, Dijon, France; 8 Meakins-Christie Laboratories, Department of Medicine, Faculty of Medicine, McGill University, Montréal, QC, Canada; 9 Interdepartmental Division of Critical Care Medicine and Department of Physiology, University of Toronto, Toronto, ON, Canada; 10 Division of Respirology, Department of Medicine, University Health Network, Toronto, ON, Canada; 11 Department of Surgery and Department of Laboratory Medicine and Pathobiology, University of Toronto, ON, Canada; 12 Faculty of Kinesiology and Physical Education, University of Toronto, Toronto, ON, Canada; 13 Banting and Best Diabetes Centre, University of Toronto, Canada; 14 Groupe de Recherche en Activité Physique Adaptée, Faculté des Sciences, Université du Québec À Montréal, Montréal, Qc, Canada

**Keywords:** Skeletal muscle, Muscle atrophy, Mitochondria, Respiration, Oxidative phosphorylation, Endurance training

## Abstract

The transcriptional repressor B cell lymphoma 6 (BCL6) is highly expressed in skeletal muscle. Although transcriptome-wide studies have shown BCL6 dysregulation in muscular dystrophies, investigations into its endogenous roles in muscle biology remain scarce. We therefore generated skeletal muscle-specific *Bcl6* knockout (M-*Bcl6* KO) mice and used adeno-associated virus to knockdown (KD) *Bcl6* selectively in limb muscles of mice. In both models, *Bcl6* deficiency led to reduced muscle mass and contractility. Single-nucleus RNA sequencing and biochemical analyses revealed upregulation of *Socs2,* and inhibition of the IGF1/AKT pathway. Mitochondrial respiration was significantly reduced in permeabilized myofibers upon *Bcl6* KO and KD, and electron microscopy showed decreased mitochondrial density and altered morphology. Pathways regulating mitochondrial quality control were also downregulated. While Bcl6 KO did not significantly impair baseline treadmill running capacity, it blunted the adaptive response to endurance training. These findings demonstrate that *Bcl6* is a critical regulator of skeletal muscle mass and mitochondrial bioenergetics, acting through transcriptional control of signaling and metabolic pathways essential for the maintenance of muscle mass and function.

## Highlights

• *Bcl6* is most highly expressed in skeletal muscle and essential for its function and mass.
• *Bcl6* is essential for maintening mitochondrial morphology and bioenergetics.
• *Bcl6* is important in the adaptive response to endurance training.
• Loss of *Bcl6* upregulates *Socs2* and alters the IGF1/AKT pathway in skeletal muscle.

## 1 Introduction

Skeletal muscle is the most abundant tissue in the human body and is essential for a broad range of physiological functions, including postural control, balance, locomotion, and breathing, as well as thermogenesis and the regulation of systemic metabolic homeostasis [1]. Given its central role in whole-body physiology, it is not surprising that skeletal muscle mass is a robust predictor of longevity and overall health [2,3]. Skeletal muscle atrophy, defined as loss of muscle mass, is a hallmark of numerous pathological or deleterious conditions, including muscular dystrophy, sepsis, cancer cachexia, immobilization, and aging [[4], [5], [6]]. Despite its profound clinical relevance, the molecular mechanisms governing the maintenance of skeletal muscle mass and function remain incompletely understood, contributing to the current lack of effective therapeutic strategies to prevent or reverse muscle wasting [7,8].

Recent studies have positioned the transcriptional repressor B-cell lymphoma (BCL6), best known for its regulatory role in humoral immune response and its oncogenic role in lymphoma [9], as a potential regulator of skeletal muscle physiology. Interestingly, this gene is most highly expressed in skeletal muscle; however, investigations into the role played by *BCL6* in skeletal muscle health and disease remain limited [10]. *In vitro* studies have demonstrated that BCL6 contributes to myogenic differentiation by inhibiting apoptosis in differentiating myoblasts and facilitating cell cycle exit to promote terminal differentiation [[11], [12], [13], [14]]. BCL6 was also reported as a critical regulator of glucose homeostasis and lipid metabolism in metabolically active tissues [[15], [16], [17], [18]], including the liver and adipose tissue. Recent studies have shown that muscle-specific *Bcl6* knockout in mice causes muscle wasting during growth and development, primarily through the dysregulation of anabolic and catabolic pathways [19,20]. *Bcl6* knockout was also associated with transcriptional downregulation of genes involved in various metabolic pathways [19,20], suggesting mitochondrial maladaptation in response to *Bcl6* deficiency. However, whether BCL6 regulates mitochondrial function remains unexplored.

To address this knowledge gap, we generated a skeletal muscle-specific *Bcl6* knockout (M-*Bcl6* KO) mouse model and used adeno-associated virus (AAV) to knock down (KD) *Bcl6* in adult mice. In line with recently published studies [19,20], we report that *Bcl6* KO causes substantial muscle atrophy and weakness, likely resulting from upregulation of *Socs2* and subsequent inhibition of the IGF-1/AKT signalling pathway. Strikingly, our investigations revealed that *Bcl6* KO and KD result in significant alterations in mitochondrial bioenergetics. Analyses of transmission electron micrographs revealed reduced mitochondrial density and altered mitochondrial morphology in M-*Bcl6* KO mice, alongside alterations in pathways regulating mitochondrial quality control. Additionally, while baseline treadmill running capacity was unaffected in Bcl6 KO mice, the absence of Bcl6 substantially impaired their ability to mount a normal endurance-training adaptation. These findings corroborate recent reports identifying BCL6 as a central regulator of skeletal muscle mass and extend current knowledge by revealing its previously unrecognized role in modulating mitochondrial bioenergetics.

## 2 Results

### 2.1 *BCL6* is highly expressed in skeletal muscle tissue

**Figure 1:**
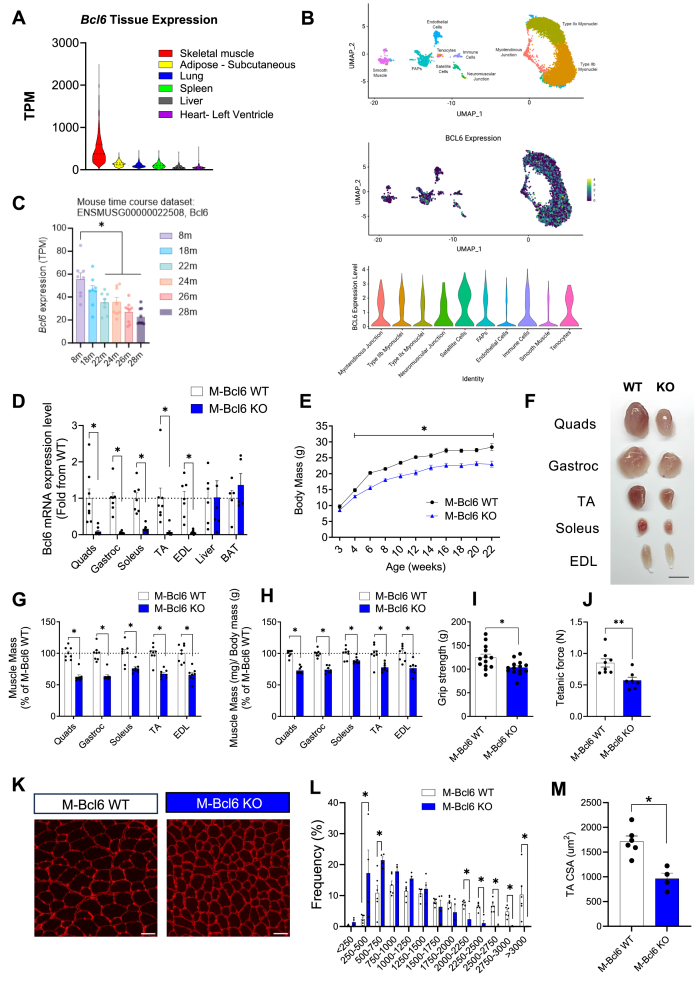
***BCL6* is highly expressed in human and mouse skeletal muscle and is essential for regulating muscle mass and function. (A)** RNA-seq data for *BCL6* tissue-specific expression pattern in various human tissues obtained from GTEx database. Expression values are shown in transcripts per million (TPM). **(B)** Unbiased clustering of snRNA-seq data of 5-month-old TA muscle represented on a UMAP (upper panel). UMAP (middle panel) and violin plots (lower panel) showing *Bcl6* gene expression for different cell populations. Data obtained from MYOATLAS database. Expression values are shown as a probability distribution across clusters. **(C)** Expression pattern of *Bcl6* from the gastrocnemius of old wild-type mice at various time points obtained from SarcoAtlas database. Expression values are shown in transcripts per million (TPM). **(D)** Quantification of *Bcl6* mRNA expression in various muscles and non-muscle tissue from 10 to 12-week-old mice assessed by RT-qPCR. Data was normalized by the housekeeping gene *36B4* and expressed as fold change compared to M-*Bcl6* WT. *n* = 4–9 per group. **(E)** Body mass of M-*Bcl6* WT and KO male mice at the indicated ages. *n* = 4–9 per group. **(F)** Representative images of the various muscle groups from the M-*Bcl6* WT (left) and M-*Bcl6* KO (right) mice at 10–12 weeks of age. Scale bar = 0.5 cm. **(G)** Tissue weights of various muscles from male M-*Bcl6* WT and M-*Bcl6* KO mice at 10–12 weeks of age. Tissue weights are shown as percent of those from M-*Bcl6* WT. *n* = 8 per group. **(H)** Tissue weights of various muscles from male M-*Bcl6* WT and KO mice normalized to body mass at 10–12 weeks of age. Normalized data are shown as percent of M-*Bcl6* WT. *n* = 8 per group. **(I)** Absolute forelimb grip strength measured in male M-*Bcl6* WT and M-*Bcl6* KO mice at 8 weeks of age. *n* = 13–14 per group. **(J)** Absolute tetanic force of gastrocnemius muscle of male M-*Bcl6* WT and KO mice at 12–15 weeks of age. *n* = 7–8 per group. **(K)** Representative images of laminin staining of the TA muscle of male M-*Bcl6* WT and KO mice, Scale bar = 50 μm; and **(L)** quantification of their myofiber cross sectional area (CSA) and **(M)** their average CSA. *n* = 4–6 per group. Data in D, E, G, H and L were analyzed with two-way ANOVA and corrections for multiple comparisons were performed with the two-stage step-up method of Benjamini, Krieger, and Yekutieli (∗p < 0.05 and q < 0.1). Data in I, J, and M were analyzed with unpaired two tailed Student's *t-*test (∗p < 0.05 and ∗∗p < 0.01). Data are presented as mean ± SEM (with individual data points).

We first examined *Bcl6* gene expression across various human tissues. Data from the Genotype-Tissue Expression (GTEx) Portal (https://gtexportal.org/home/) revealed that *Bcl6* expression is significantly higher in skeletal muscle compared to other tissues, including adipose tissue, lung, spleen, liver, and heart (Figure 1A). Given the complexity of muscle tissue, we next analyzed a published dataset from MyoAtlas (https://research.cchmc.org/myoatlas/) [21] of snRNA-seq from mouse tibialis anterior (TA) muscle to identify which nuclei within the muscle tissue express *Bcl6.* The MyoAtlas snRNA-seq data revealed that *Bcl6* is expressed in all cell types present in muscle tissues, including type IIX and IIB fibers, tenocytes, as well as satellite cells, fibro-adipogenic progenitors, endothelial cells, immune cells, and smooth muscle cells (Figure 1B). In myofibers, *Bcl6* is also expressed in myonuclei present at the neuromuscular and myotendinous junctions (Figure 1B). Among these, the highest expression was observed in satellite cells, while the lowest was detected in smooth muscle cells. Taken together, these gene expression data indicate that *Bcl6* is highly expressed in skeletal muscles, suggesting that BCL6 may be important for muscle homeostasis. We next assessed *Bcl6* expression in skeletal muscle during aging using high-throughput datasets obtained from SarcoAtlas (https://sarcoatlas.scicore.unibas.ch/) [22], which demonstrates that *Bcl6* expression progressively decreases with aging throughout the lifespan in mice (Figure 1C). This observation indicates that *Bcl6* expression is downregulated in aged skeletal muscle, similar to other models of muscle atrophy, as shown in transcriptome-wide studies that report reduced *Bcl6* expression in the muscles of cancer cachexia mouse models [23,24].

### 2.2 Mice with skeletal muscle-specific *Bcl6* deficiency have reduced body mass, muscle mass and strength without defects in glucose homeostasis

To investigate the *in vivo* role of *Bcl6* in skeletal muscle, we generated a muscle-specific-*Bcl6* knockout (M-*Bcl6* KO) mouse model using the Cre-LoxP system under control of the *Myf6* promoter. We have previously reported that this *Cre* transgene is exclusively expressed in skeletal muscle cells [25]. To ensure efficient gene deletion, we used *Bcl6* floxed mice with loxP sites that flank exons 7–9 of the *Bcl6* gene, which contains the zinc-finger motifs responsible for DNA binding [26]. To verify the specificity of *Bcl6* deletion in muscle, we quantified *Bcl6* expression by RT-qPCR in multiple tissues. As shown in Figure 1D, *Bcl6* mRNA was efficiently deleted selectively in skeletal muscles, including the quadriceps (QUAD), gastrocnemius (GAS), soleus, extensor digitorum longus (EDL), and tibialis anterior (TA), but not in the liver. Also, *Bcl6* was not deleted in brown adipose tissue (BAT), which shares common lineage to skeletal muscle during development [27], further demonstrating tissue-specific deletion (Figure 1D).

M-*Bcl6* KO mice were born in Mendelian ratio and appeared normal at birth, with their body mass similar to littermate controls, suggesting no defects during development (Figure 1E and Supplemental 1A). However, as early as 4 weeks of age, their body mass was significantly lower compared to age- and sex-matched control mice (Figure 1E and Supplemental 1A). This lower body mass persisted throughout adulthood, where the difference compared to control littermates was maintained without apparent progression. This reduction in body mass was sustained to 1 year of age in both male and female M-*Bcl6* KO mice (Supplemental 1B–C). Given the importance of skeletal muscle in glucose homeostasis as a major site of glucose disposal, we explored whether loss of *Bcl6* impacted glucose and insulin tolerance. Interestingly, there were no significant differences in glucose or insulin tolerance in 15-week-old M-*Bcl6* KO male and female mice compared to their control littermates (Supplemental 1D-G). Furthermore, these analyses even at 1 year of age revealed no major differences between M-*Bcl6* KO mice and WT mice in both sexes (Supplemental 1H–K). These findings suggest that loss of *Bcl6* does not result in major alterations in whole-body glucose homeostasis.

Along with their lower total body mass, both male and female M-*Bcl6* KO mice exhibited visibly smaller hindlimb muscle groups (Figure 1F) with a significant decrease in muscle mass as compared to littermate controls (Figure 1G and Supplemental 2A). This decline in muscle mass persisted even after normalization to total body mass (Figure 1H and Supplemental 2B). This atrophic phenotype was seen across all muscle groups, with M-*Bcl6* KO mice displaying a lower muscle mass for the Quads, GAS, soleus, TA and EDL muscles. The weights of other tissues, including the heart, liver, lungs, pancreas, or epididymal adipose tissue were comparable to those of littermate controls, indicating that muscle *Bcl6* did not have any major effect outside of the tissue in which the gene was deleted (Supplemental 2C-D). The loss of muscle mass was also evident at 1 year of age in both male and female M*-Bcl6* KO mice (Supplemental 2E-F).

In line with data indicating muscle atrophy, M-*Bcl6* KO mice displayed lowered grip strength as compared to sex-matched control mice (Figure 1I and Supplemental 2G). These differences in grip strength were not present when normalized to body mass (Supplemental 2H–I), indicating that muscle weakness caused by *Bcl6* deletion is likely primarily driven by muscle atrophy rather than intrinsic muscle contractile dysfunction. Muscle atrophy-associated weakness persisted in both male and female M-*Bcl6* KO mice that aged to 1 year (Supplemental 2J-K). Similarly, the *in vivo* force generation of the GAS muscle was decreased by ∼32% in male M-*Bcl6* KO mice compared to WT mice (Figure 1J). As well, histological analysis of TA muscle sections revealed that M-*Bcl6* KO mice had a greater proportion of small fibers and a lower proportion of large fibers *vs* M-*Bcl6* WT controls (Figure 1KandL). BCL6-deficient muscle displayed a ∼40% lower myofiber mean cross-sectional area (CSA) compared to M-*Bcl6* WT controls (Figure 1M). These results collectively demonstrate that muscle-specific *Bcl6* deletion induced significant myofiber atrophy.

### 2.3 AAV-mediated *Bcl6* knockdown in adult mice reduces muscle mass and contractility

**Figure 2:**
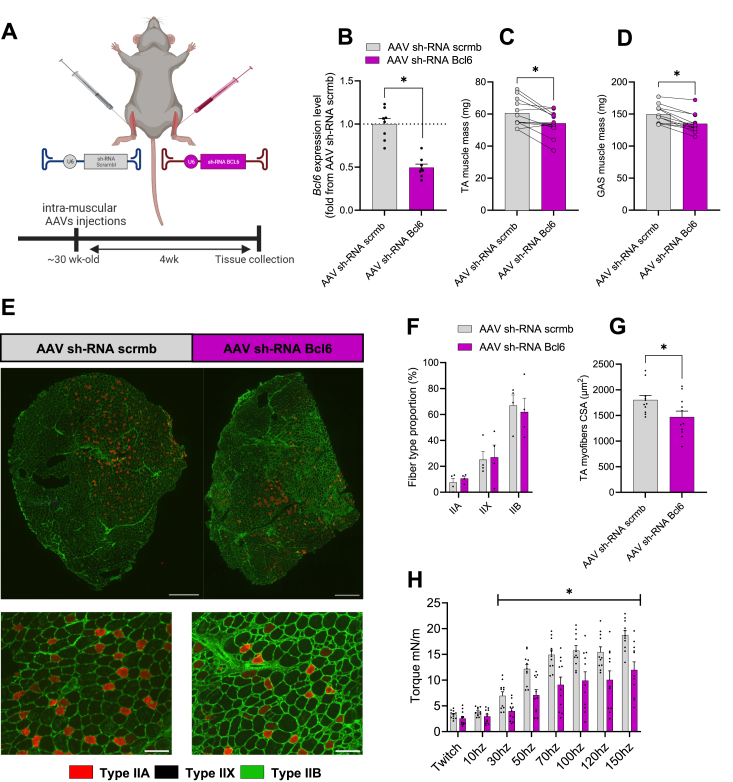
**AAV-KD of *Bcl6* in adult mice causes muscle atrophy and weakness. (A)** Schematic representation of experimental design created with BioRender.com. **(B)** Quantification of *Bcl6* mRNA expression from male mice injected with either AAV-scrmb or AAV-sh*Bcl6* knockdown (KD) in tibialis anterior (TA) muscle assessed by RT-qPCR. *n* = 7–8 per group. **(C)** Tissue mass of TA muscle from male mice injected with either AAV-scrmb and AAV-sh *Bcl6* KD muscle. *n* = 8 per group. **(D)** Tissue mass of gastrocnemius (GAS) muscle from male mice injected with either AAV-scrmb and AAV-sh*Bcl6* KD muscle. *n* = 8 per group. **(E)** Representative myosin heavy chain (MHC) immunolabelling of fiber type proportion in TA muscle of male mice injected with either AAV-scrmb or AAV-sh*Bcl6* KD. Large Scale bars = 200 μm, inset scale bar = 50 μm. **(F)** Analysis of fiber type proportion in TA muscle of male mice injected with either AAV-scrmb or AAV-sh*Bcl6* knockdown. *n* = 4–5 per group. **(G)** Average cross-sectional area (CSA) of TA muscle of male mice injected with either AAV-scrmb or AAV-sh*Bcl6* knockdown. *n =* 11 per group. **(H)***In vivo* isometric plantarflexor torque measured at 4 weeks post-AAV injection. *n* = 11 per group. Data in B, C, D, F and G were analyzed with paired two-tailed Student's *t*-test (∗p < 0.05). Data in H was analyzed with two-way ANOVA and corrections for multiple comparisons were performed with the two-stage step-up method of Benjamini, Krieger, and Yekutieli (∗p < 0.05 and q < 0.1). Data are presented as mean ± SEM (with individual data points).

We next investigated whether adeno-associated virus (AAV)-mediated acute knockdown (KD) of *Bcl6* in adult mice would also alter muscle mass and function. Using the contralateral limb as an endogenous control, we determined the effects of AAV-mediated acute KD of *Bcl6* in TA and GAS muscles of 30-week-old mice (Figure 2A). RT-qPCR analysis confirmed that our approach resulted in ∼50% KD in *Bcl6* gene expression in TA muscle at 4 weeks post-injection (Figure 2B). Remarkably, and in line with data shown in M*-Bcl6* KO mice, the TA and GAS masses were lower in *Bcl6-*KD muscles compared to AAV-scrmb injected muscles (Figure 2B–D). Given that myofiber type composition influences both contractile and metabolic properties, we next examined fiber type distribution in TA muscles using a triple myosin heavy chain (MHC) immunolabelling on muscle cross-sections (Figure 2E). Notably, acute KD of *Bcl6* did not alter the proportion of type IIB, IIX, and IIA myofibers (Figure 2F), indicating unaltered myofiber composition. However, consistent with the muscle atrophy seen in M-*Bcl6* KO mice, knockdown of *Bcl6* also resulted in a significant decrease (∼18%) in myofiber CSA in TA muscles, compared to AAV-scrmb injected muscles (Figure 2G). We next measured the effects of *Bcl6*-KD on muscle function *in vivo* by measuring the force generated by the hindlimb dorsiflexor muscles in anesthetized mice using electrical stimulation via surface electrodes. Consistent with our observation in M-*Bcl6* KO mice, we found a significant reduction in *in vivo* isometric torque production in *Bcl6*-KD muscles (Figure 2H). Together, these data reveal that even acute KD of *Bcl6* in fully developed normal adult muscle in an inducible manner is sufficient to cause muscle atrophy and weakness, highlighting the critical role of *Bcl6* in the maintenance of muscle homeostasis.

### 2.4 *Bcl6*-deficiency alters pathways regulating protein synthesis and degradation

**Figure 3:**
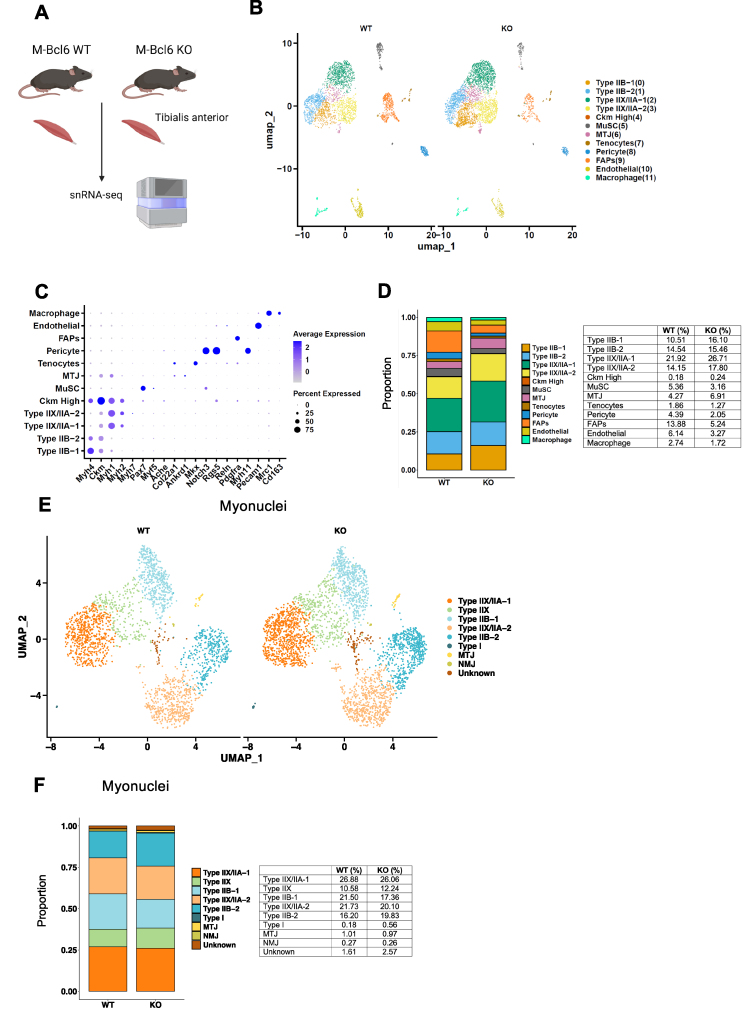
**Single-nucleus RNA sequencing shows no significant changes to fibre type composition of muscles with *Bcl6* KO. (A)** Schematic representation of experimental design created with BioRender.com. **(B)** Unbiased clustering showing nuclear transcriptomes of snRNA-seq data in 6-week-old M-*Bcl6* WT (left) and M-*Bcl6* KO (right) TA muscle represented on a UMAP, colored by cluster identity. **(C)** Dot plot from snRNA-seq showing gene markers used for cluster identification. Darker-colored dots indicate higher expression, and larger dots indicate a greater percentage of nuclei expression of the corresponding gene. **(D)** Bar graph showing proportions (left) and percentages (right) of the composition of nuclei showing myofibre and cell type in M-*Bcl6* WT and KO TA muscle. **(E)** Unbiased clustering of snRNA-seq data for myonuclear population in M-*Bcl6* WT (left) and KO (right) TA muscle represented on a UMAP, colored by cluster identity. **(F)** Bar graph showing proportions (left) and percentages (right) of the composition of myonuclei indicating fibre type in M-*Bcl6* WT and KO TA muscle. The right and left TA muscles from 2 male mice were used per group.

To uncover the transcriptional changes driving muscle atrophy in BCL6-deficient muscle, we performed single-nucleus RNA sequencing (snRNA-seq) in TA muscle from M-*Bcl6* KO and M-*Bcl6* WT mice (Figure 3A). After quality control, we retained a total of 6921 nuclei, including 3320 nuclei from control group and 3601 nuclei from M-*Bcl6* KO muscles. The identity of each nuclear cluster was determined by the expression of specific genes, as previously reported [21]. Using unsupervised Uniform Manifold Approximation and Projection (UMAP) plots, we further illustrated the cellular heterogeneity with 12 distinct subclusters within the skeletal muscle from M-*Bcl6* WT and M-*Bcl6* KO mice (Figure 3B–C). No striking change in nuclear composition was observed between M-*Bcl6* KO and WT muscles. (Figure 3D). When only myonuclei were considered, no noticeable differences were observed (Figure 3E–F), suggesting that Bcl6 deficiency does not substantially alter fiber type composition. In line with muscle atrophy in M-*Bcl6* KO and WT muscles, gene ontology revealed that muscle tissue development ranked in the top downregulated biological processes in all myonuclear fractions (Supplemental Fig. 3A–D).

**Figure 4:**
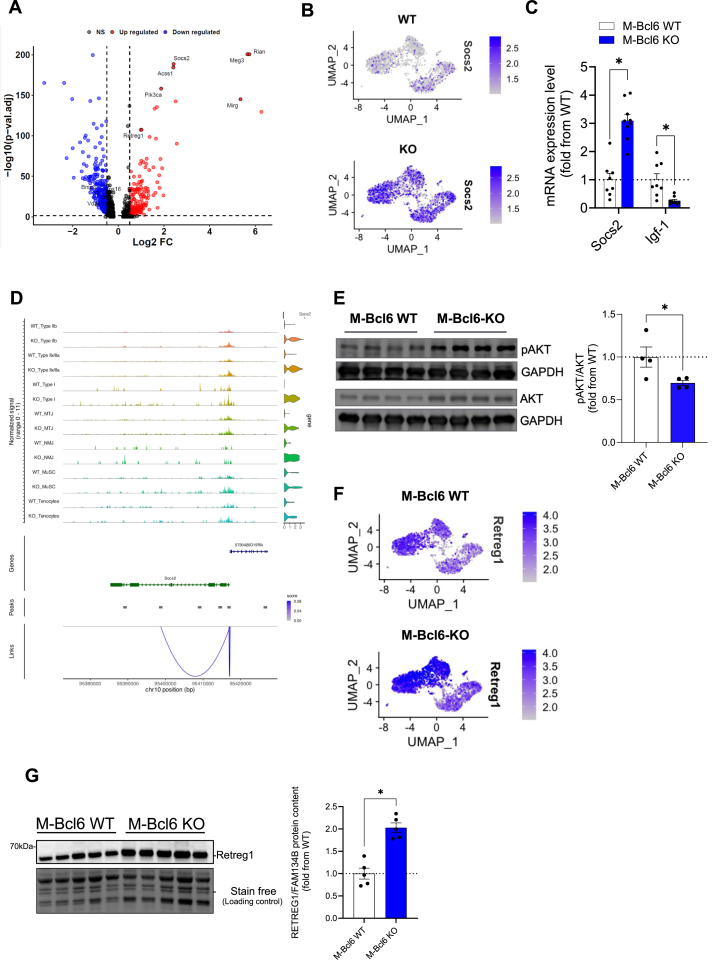
***Bcl6* regulates *Socs2* expression, the IGF1/AKT signalling pathway and proteostasis: (A)** Volcano plot showing upregulated (red) and downregulated (blue) genes in M-*Bcl6* KO vs M-*Bcl6* WT male mice from snRNA-seq data. (**B**) UMAP plot of the myonuclear gene expression of *Socs2* in 6-week-old male M-*Bcl6* WT (top) and M-*Bcl6* KO (bottom) mice derived from snRNA-seq data. (**C**) Quantification of *Socs2* and *Igf1* mRNA expression in quadriceps of 10–12-week-old male M-*Bcl6* WT and M-*Bcl6* KO mice obtained by RT-qPCR. mRNA expression levels are shown relative to housekeeping gene β-actin and are expressed as fold change compared to M-*Bcl6* WT. *n* = 8–9 per group. (**D**) snATAC-seq data showing increased chromatin accessibility for *Socs2* in 6-week-old male M-*Bcl6* KO mice *vs* M-*Bcl6* WT. (**E**) Immunoblot detection (left) of AKT and p-AKT from TA muscles and corresponding quantification (right) of the p-AKT/AKT ratio in 10–12-week-old male M-*Bcl6* WT and M-*Bcl6* KO mice. *n =* 4 per group. (**F**) UMAP plot of the myonuclear gene expression of *Retreg1* (also known as Fam134b) in 6-week-old male M-*Bcl6* WT (top) and M-*Bcl6* KO (bottom) mice derived from snRNA-seq data. (**G**) Immunoblot detection (left) of Retreg1 from quadriceps of 10–12-week-old male M-*Bcl6* WT and KO mice and corresponding quantification (right). *n* = 5 per group. Data in C, F and H were analyzed with paired two-tailed Student's *t*-test (∗p < 0.05). Data are presented as mean ± SEM (with individual data points).

In line with the known role of *Bcl6* as a transcription repressor of Socs2 [28], we found that Socs2 was one of the most upregulated genes in response to *Bcl6* KO (Figure 4A–C). snATAC-seq data confirmed the increased accessibility of Socs2 in response to *Bcl6* KO (Figure 4D). In line with its role as a negative regulator of GH/IGF1 signalling, this increase in Socs2 expression was associated with a decrease in the expression of IGF1 (Figure 4C) and a decrease in PI3K signaling as shown by lower pAKT/AKT ratio (Figure 4E). Similar to previous observations [19], *Retreg1,* a key regulator of ER-phagy [29] that has been shown to be upregulated in various atrophy conditions [30], was also upregulated at the mRNA and protein levels in *Bcl6* KO muscle (Figure 4A, F-G). Taken altogether, these data show Bcl6 as a critical regulator of Socs2 and of the IGF1/AKT signalling pathway, a pathway well known for its key role in muscle mass regulation [31]. Other notable upregulated genes included three long noncoding RNA located in the Dlk1-Dio3 locus, namely *Meg3*, *Rian* and *Mirg* (Figure 4A and Supplemental Fig. 4A–F), suggesting a potential regulatory role of *Bcl6* in select lncRNAs. Notable downregulated genes in M-*Bcl6* KO muscle included the mitochondrial proteins *Vdac1*, *Cox16* and *Bnip3* (Figure 4A). Since mitochondria are essential to the maintenance of skeletal muscle health [32,33], these findings prompted us to investigate whether *Bcl6* could regulate mitochondrial content and function.

### 2.5 *Bcl6-*deficiency in skeletal muscles alters mitochondrial content and ultrastructure

**Figure 5:**
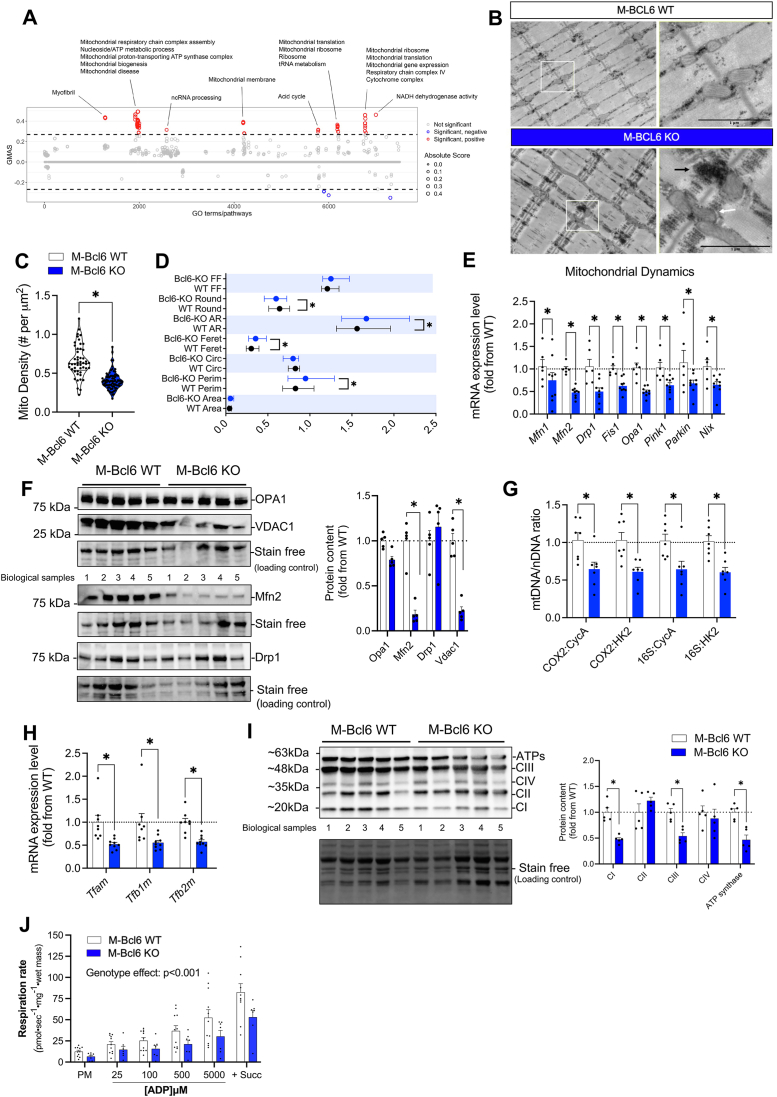
**Ultrastructural abnormalities and mitochondrial dysfunction in *Bcl6* KO muscle. (A)** Manhattan plot of ontology terms and pathways associated with *Bcl6* gene function in skeletal muscle tissue. All terms significantly associated with *Bcl6* expression in muscle tissue (GMAS ≥0.268, <=-0.268) are highlighted. Data obtained from GeneBridge database. **(B)** Electron micrographs of M-*Bcl6* WT (upper panel) and M-*Bcl6* KO (lower panel) gastrocnemius (GAS) muscle at 10–12 weeks of age. Scale bar = 1 μm. Black arrows represent glycogen accumulation and white arrow represents altered mitochondria morphology. **(C)** Quantification of mitochondrial density analyzed from electron micrographs of M-*Bcl6* WT and KO GAS muscle. **(D)** Mean quantification of morphometric and shape descriptors of intermyofibrillar mitochondria, including Form Factor (FF), Roundness, Aspect Ratio (AR), Feret's diameters (Feret), Circularity (Circ), Perimeter (Perim), and Area in male M-*Bcl6* WT and KO mice. **(E)** Quantification of genes involved in mitochondrial quality control processes in quadriceps of 10-week-old male M-*Bcl6* WT and KO mice. mRNA expression levels are shown relative to housekeeping gene β-actin and are expressed as fold change compared to M-*Bcl6* WT as assessed by RT-qPCR. *n* = 6–9 per group. **(F)** Immunoblot detection (right) and corresponding protein quantification (left) of OPA1, VDAC1, Mfn2, and Drp1 in quadriceps of male M-*Bcl6* WT and M–KO mice. *n* = 5 per group. **(G)** Mitochondrial DNA (mtDNA) copy number calculated as a ratio of mitochondrial encoded genes (*Cox2* or *16S*) to nuclear encoded genes (*CycA* or *HK2*) as measured by RT-qPCR from quadriceps of 12-week-old M-*Bcl6* WT and M-*Bcl6* KO mice. *n* = 7 per group. **(H)** Quantification of mitochondrial biogenesis genes in quadriceps of 10-week-old male M-*Bcl6* WT and KO mice. mRNA expression levels are shown relative to housekeeping gene β-actin and are expressed as fold change compared to M-*Bcl6* WT assessed by RT-qPCR. *n* = 8–9 per group. **(I)** Immunoblot detection (left) and corresponding protein quantification (right) of representative subunits of the mitochondrial oxidative phosphorylation (mtOxPhos) from quadriceps of male M-*Bcl6* WT and M-*Bcl6* KO mice. *n* = 5 per group. (**J**) Mitochondrial respiration of male and female M-*Bcl6* WT and M-*Bcl6* KO mice performed by the addition of pyruvate and malate (PM) in permeabilized muscle fibers across a range of ADP concentrations, and succinate (Succ) in the presence of creatine. *n* = 7–11 per group. Data in C, D, E, F, H, and I were analyzed by unpaired two-tailed Student's *t*-test (∗p < 0.05). Data in J was analyzed with two-way ANOVA, and corrections for multiple comparisons were performed with the two-stage step-up method of Benjamini, Krieger, and Yekutieli (∗p < 0.05 and q < 0.1). Data are presented as mean ± SEM (with individual data points).

To further investigate the impact of *Bcl6* disruption in skeletal muscle, we explored biological pathways and gene ontology terms associated with its gene function. Using GeneBridge (https://systems-genetics.org) [34], we interrogated in an unbiased manner the role of *Bcl6* in skeletal muscle of mice by identifying key functional associations and potential regulatory networks. The Manhattan plot for gene sets associated with *Bcl6* function revealed numerous mitochondrial-related pathways, as highlighted in Figure 5A. These findings suggest a role for *Bcl6* in the regulation of mitochondrial biology in skeletal muscles. We next assessed the impact of muscle-specific *Bcl6* KO on mitochondrial morphology and ultrastructure using transmission electron microscopy (TEM) (Figure 5B). Analyses of TEM images revealed that M-*Bcl6* KO muscle displayed a lower mitochondrial density in comparison to littermate controls (Figure 5C). Additionally, assessment of morphometric parameters from TEM images in the intermyofibrillar regions revealed that M-*Bcl6* KO mice have increased mitochondrial perimeter, Feret's diameter and aspect ratio, and decreased circularity and roundness (Figure 5D). These morphometric changes are remarkably similar to those reported in glycolytic fibers that occur with aging in rodents [35,36]. These findings suggest that the lower muscle mass and dysfunction that are present in *Bcl6* KO muscle may represent accelerated aging defects (Figure 1C) that may be contributed by mitochondrial changes that are associated with sarcopenia. These findings indicate that *Bcl6* is required to maintain normal mitochondrial content and morphology.

Consistent with the quantitative EM analysis, the lack of *Bcl6* in muscle was accompanied by a marked reduction in the mRNA expression levels of key genes related to mitochondrial dynamics (*Mfn1*, *Mfn2*, *Drp1*, *Fis1*, *Opa1*) and mitophagy (*Pink1*, *Park2* and *Nix*) (Figure 5E). In line with these findings, immunoblotting further revealed that Bcl6 deficiency resulted in a decrease in the protein levels of mitofusin 2 (Mfn2) and voltage-dependent anion channel 1 (VDAC1), while no significant change was observed for the protein levels of OPA1 and Drp1 (Figure 5F). We next assessed mitochondrial DNA (mtDNA) content by quantifying the mtDNA-to-nuclear DNA (nDNA) ratio using RT-qPCR. Using various primer sets (COX2:CyA, COX2:HK2, 16S:CyA, and 16S:HK2), we consistently found that the mtDNA/nDNA ratio was lower in M-*Bcl6* KO muscles, further supporting reduced mitochondrial content (Figure 5G). In addition, we observed reduced gene expression of key regulators of mitochondrial biogenesis, including mitochondrial transcription factor A (*Tfam*), B1 (*Tfb1m*) and B2 (*Tfb2m*) (Figure 5H). Immunoblotting revealed reduced protein content of the representative subunits of Complex I, Complex IV and ATP synthase in the GAS from M-*Bcl6* KO mice (Figure 5I), further supporting that mitochondrial content is decreased in the absence of BCL6.

To assess whether these changes in mitochondrial parameters translated into their altered function, we next assessed the impact of M-*Bcl6* KO on mitochondrial respiration in permeabilized myofibers obtained from the GAS muscle, using high resolution respirometry. As shown in Figure 5J, M-*Bcl6* KO muscle fibres displayed significantly lower mitochondrial respiration *vs* those of WT mice. This decrease in mitochondrial respiration was also associated with an increase in glycogen content (Supplemental Fig. 5A). Since this increase in glycogen can result from altered substrate handling that may favor lipid accumulation, we also measured triglyceride content in the GAS muscle, which did not demonstrate any difference between M-*Bcl6* WT and M-*Bcl6* KO mice (Supplemental Fig. 5B). These data show that loss of muscle *Bcl6* does not alter intramyocellular lipid content.

**Figure 6:**
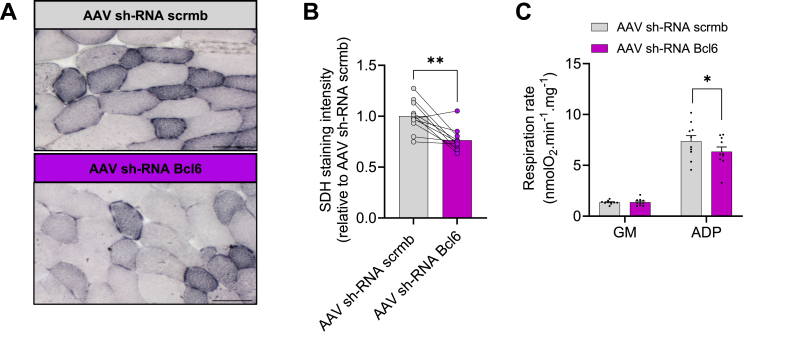
**AAV-KD of *Bcl6* in adult mice reduces mitochondrial content and respiration**. **(A)** Representative images of succinate dehydrogenase (SDH) histochemistry of TA muscle 4 weeks post AAV-injection. Scale bar = 50 μm. **(B)** Quantification of SDH staining from TA muscle injected with either AAV-scrmb or AAV-sh*BCL6* knockdown. *n =* 10 per group. **(C)** Mitochondrial respiration of GAS muscle injected with either AAV-scrmb or AAV-sh*BCL6* knockdown. GM: respiration rate driven by the addition of Glutamate and Malate. ADP: respiration rate driven by the subsequent addition of ADP. *n* = 10 per group. Data in B were analyzed with paired two-tailed Student's *t*-test (∗∗p < 0.01). Data in C were analyzed with two-way ANOVA, and corrections for multiple comparisons were performed with the two-stage step-up method of Benjamini, Krieger, and Yekutieli (∗p < 0.05 and q < 0.1). Data are presented as mean ± SEM (with individual data points).

We next assessed whether *Bcl6-*KD can lead to mitochondrial dysfunction. As shown in Figure 6, *Bcl6-*KD resulted in a decrease in succinate dehydrogenase (SDH) expression, a marker of mitochondrial content and oxidative capacity [37], measured *in situ* on muscle cross-sections (Figure 6A–B). These data show that *Bcl6* KD may be sufficient to alter mitochondrial function. To explore this further, we next examined whether *Bcl6* KD altered mitochondrial respiration in permeabilized myofibers bundles prepared from the GAS muscle. Consistent with the reduction in SDH expression, we observed a significant decrease in mitochondrial respiration (state III; ADP-stimulated) driven by complex I substrates (glutamate & malate) in muscles with *Bcl6* KD (Figure 6C).

Collectively, our data indicate that *Bcl6* deficiency in skeletal muscle causes a wide spectrum of ultrastructural abnormalities and alters mitochondrial content, structure and function. These findings are present both in M-*Bcl6* KO as well as after inducible KD, highlighting the critical role of *Bcl6* in the maintenance of muscle mass and function that are likely related to its homeostatic role in mitochondrial function.

### 2.6 *Bcl6-*deficiency in skeletal muscle blunted adaptation to endurance training

**Figure 7:**
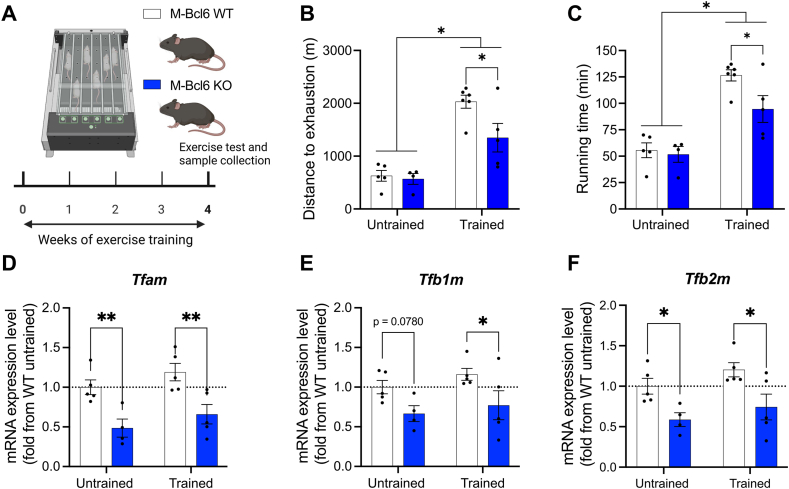
***Bcl6* KO mice display blunted adaptation to endurance training**. **(A)** Schematic representation of experimental design created with BioRender.com. **(B–C)** Distance **(B)** and time **(C)** achieved by untrained and trained M-*Bcl6* WT and M-*Bcl6* KO mice during a treadmill exhaustion test. **(D–F)** Quantification of mitochondrial biogenesis genes (D-*Tfam*, E-*Tfb1m* and F-*Tfb1m*) in the quadriceps of untrained and trained male M-*Bcl6* WT and KO mice. mRNA expression levels are shown relative to housekeeping gene *18s* and are expressed as fold change compared to M-*Bcl6* WT assessed by RT-qPCR. Data were analyzed by two-way ANOVAs followed by a Fisher's LSD post hoc test (∗p < 0.05). Data are presented as mean ± SEM (with individual data points).

Based on our findings that BCL6 deletion impairs mitochondrial bioenergetics, we next examined whether Bcl6 deficiency alters skeletal muscle adaptation to endurance training. After a 4-week endurance training protocol (Figure 7A), both M-*Bcl6* WT and M-*Bcl6* KO mice demonstrated an improvement in running performance compared with their untrained counterpart (Figure 7B–C). Although baseline treadmill running capacity did not differ between genotypes, M-*Bcl6* KO mice displayed a markedly blunted training-induced increase in total running time and distance to exhaustion (Figure 7B–C). Genes involved in mitochondrial biogenesis, including *Tfam*, *Tfb1m*, and *Tfb2m*, were downregulated at baseline in M-*Bcl6* KO mice. While M-*Bcl6* WT showed a clear trend in the upregulation of these genes after training, this training-induced upregulation appeared blunted in M-*Bcl6* KO mice (Figure 7D–F).

## 3 Discussion

While *BCL6* is well known to play key roles in humoral immunity [9], B cell development [38], and oncogenic signalling [39], investigations into its roles in skeletal muscle biology remain scarce. To address this gap, we developed skeletal muscle-specific *Bcl6* KO mice and employed AAV-mediated *Bcl6* KD in adult mice. In both models, *Bcl6* deficiency resulted in reduced muscle mass and impaired contractile function. Based on publicly available data positioning BCL6 as a potential regulator of mitochondrial function, we probed whether *Bcl6* deficiency could lead to altered mitochondrial structure and function. Transmission electron microscopy showed decreased mitochondrial density and altered morphology in M-*Bcl6* KO mice. In line with these findings, pathways involved in mitochondrial quality control were dysregulated in M-*Bcl6* KO mice. Both KO and KD models demonstrated a significant reduction in mitochondrial respiration. Despite marked muscle atrophy and reduced mitochondrial respiration, M-*Bcl6* KO mice displayed largely unaltered glucose tolerance and insulin sensitivity. While M-*Bcl6* KO mice did not display significant impairment in running capacity at baseline, they displayed blunted adaptive response to endurance training. Collectively, these findings establish Bcl6 as a key regulator of skeletal muscle mass and mitochondrial bioenergetics.

The pronounced muscle atrophy observed following *Bcl6* KO in our study aligns with reports from two independent groups [19,20]. While our approach involved targeting exons 7 to 9 of the *Bcl6* gene, previous studies have demonstrated that constitutive deletion of exons 3 and 4, as well as both constitutive and inducible excision of exon 5 in skeletal muscle, similarly result in marked muscle atrophy [19,20]. In agreement with these reports, we also identified upregulation of *Socs2*, subsequent suppression of the IGF1/Akt signaling cascade and impaired proteostasis as likely key mediators of the atrophic phenotype [19,20]. Importantly, our findings extend the current literature by demonstrating that a partial (∼50%) knockdown of *Bcl6* is sufficient to induce atrophy and weakness in mature skeletal muscle of adult mice. Given the age-associated reduction in *Bcl6* expression reported herein using the SarcoAtlas database (https://sarcoatlas.scicore.unibas.ch/) [22], this observation may hold particular clinical relevance. Age-related impairments in mitochondrial function, proteostasis, and anabolic signaling are well-established hallmarks of human sarcopenia [33,[40], [41], [42]] and are particularly pronounced in individuals who do not meet physical activity recommendations [41,43,44]. The pathways regulated by BCL6 in this study, particularly those involving mitochondrial quality control and IGF1/Akt signaling, map closely onto processes often implicated in muscle aging [33,41]. The observation that even a partial reduction in Bcl6 is sufficient to induce atrophy and mitochondrial dysfunction suggests that the decline in BCL6 expression reported herein in aging muscle may have functional consequences, highlighting the potential translational relevance of the pathways identified here. A decrease in *Bcl6* expression was also reported in several conditions affecting skeletal muscles, including Duchenne muscular dystrophy [45,46], myotonic dystrophy [47], spinal and bulbar muscular atrophy [48], and cancer cachexia (GSE65936, GSE48363) [23,24,49]. Our findings raise the possibility that *Bcl6* downregulation may contribute to several muscle-wasting conditions, warranting further investigation.

An intriguing finding from the present study is the striking upregulation of lncRNA from the *Dlk1-Dio3* locus, namely *Meg3*, *Rian* and *Mirg*. LncRNAs from this locus, and particularly *Meg3*, have recently emerged as potential regulators of myogenesis, muscle regeneration, muscle mass and function, and mitochondrial metabolism [[50], [51], [52], [53], [54]]. Whether the marked increase in the expression of these lncRNA contributed to the atrophic phenotype observed upon *Bcl6* KO, or whether these changes represented a compensatory response to mitigate atrophy, cannot be inferred from the data presented herein. However, based on a recent study that has reported muscle atrophy in response to *Meg3* depletion and muscle hypertrophy in response to *Meg3* overexpression, it is tempting to speculate that the increase in *Meg3* expression we report herein may be a compensatory response [55]. Regardless, based on the marked upregulation of lncRNAs of the *Dlk1-Dio3* locus we report in response to *Bcl6* KO and the emerging data showing that these lncRNAs likely play important roles in muscle biology, we believe that additional investigations are warranted to assess whether these can be modulated to attenuate muscle atrophy and weakness that occur in various catabolic conditions and diseases.

To date, the effects of *Bcl6* KO on mitochondrial structure and function have not been directly investigated. Previous studies have concluded that *Bcl6* KO does not elicit a major mitochondrial phenotype; however, these conclusions were primarily based on indirect assessments. For instance, Wang et al. reported transcriptional downregulation of mitochondrial-encoded OXPHOS complexes in *Bcl6* KO muscle yet paradoxically observed increased protein levels of representative OXPHOS subunits and VDAC. Additionally, they detected no significant difference in succinate dehydrogenase (SDH) activity in muscle homogenates [28]. Similarly, Ramachandran et al. found no reduction in mitochondrial DNA or VDAC content in *Bcl6* KO muscle and even noted a modest increase in a complex II subunit, with no changes in other representative OXPHOS subunits. While these findings contrast with the reductions in mitochondrial DNA and VDAC content reported herein following *Bcl6* KO, a discrepancy potentially attributable to differences in the knockout strategy, none of the aforementioned studies conducted a comprehensive analysis of mitochondrial structure and function.

In the present study, we provide robust evidence that *Bcl6* KO induces significant alterations in mitochondrial architecture and bioenergetic function. Transmission electron microscopy revealed a reduction in mitochondrial density accompanied by increased morphological complexity, as indicated by decreased roundness and circularity, and increased form factor and aspect ratio. These structural changes are associated with the downregulation of key pathways governing mitochondrial quality control, including mitochondrial biogenesis, dynamics and mitophagy. High-resolution respirometry further demonstrated that *Bcl6* KO leads to a reduction in maximal mitochondrial oxidative capacity. Supporting these findings, partial inducible knockdown of *Bcl6* (∼50%) was sufficient to impair mitochondrial respiration. Lastly, M-*Bcl6* KO mice blunted adaptation to endurance training. Collectively, these data using complementary approaches strongly demonstrate that *Bcl6* plays a critical role in maintaining mitochondrial homeostasis. Mitochondria play a central role of in preserving skeletal muscle mass and function [32,33]. Thus, M-*Bcl6* KO mice showing impairments in mitochondrial oxidative capacity and ultrastructure along with changes in many transcriptions and protein profile that are known to play a critical role in many aspects of mitochondrial biology may have contributed to their atrophic phenotype.

Despite the marked atrophy and mitochondrial impairment observed with *Bcl6* deletion, processes that are important in glucose metabolism, no overt changes in glucose homeostasis were present. These findings may reflect compensatory regulation by other metabolic tissues, such as adipose or liver in preserving whole-body glucose homeostasis despite the significant defects in *Bcl6*-deficient muscle. Consistent with our findings, other studies have shown that skeletal muscle mitochondrial dysfunction can occur without concomitant glucose intolerance [25,56]. In addition, insulin resistance has been reported despite enhanced mitochondrial oxidative capacity [57,58], and greater muscle mass does not always predict improved glucose tolerance or insulin sensitively [59].

Although the upstream regulation of BCL6 in skeletal muscle remains poorly defined, several pathways known to modulate BCL6 in other tissues may be relevant. BCL6 participates in cytokine-responsive transcriptional control through interaction with the SMRT/NCoR corepressor complex, which modulates inflammatory gene programs [60]. In germinal center B cells, activation of NF-κB signaling downregulates BCL6 expression [38,61]. BCL6 may also be subject to endocrine metabolic regulation in skeletal muscle, as growth hormone suppresses its transcription via the JAK/STAT5 pathway [20]. Although it is not yet known which, if any, of these pathways contribute to the age-related decline of BCL6 in muscle, these established regulatory inputs indicate that BCL6 integrates inflammatory and hormonal signals. This framework highlights potential physiological or pharmacological nodes that may influence BCL6 expression and offers testable hypotheses regarding how BCL6-dependent muscle maintenance may be supported during aging or disease.

In summary, the present study identifies *Bcl6* as an important regulator of skeletal muscle homeostasis. Mechanistically, our data and those from others [19,20] indicate that *Bcl6*, at least in part, regulates muscle mass by repressing *Socs2*, thereby preventing the inhibition of the IGF1/Akt signalling pathway and maintaining a normal balance between protein synthesis and degradation. Furthermore, our data highlight that *Bcl6* also plays an important protective role in the maintenance of mitochondrial content, structure and bioenergetic function. Collectively, these results indicate that *Bcl6* is an important regulator of muscle and mitochondrial homeostasis. Whether *Bcl6* can be modulated to attenuate muscle atrophy and impaired mitochondrial function in various disorders will require further investigation.

## 4 Materials and methods

### 4.1 Experimental model and subject details

#### 4.1.1 Animals

All experiments involving animals were approved by the Toronto General Hospital Research Institute's Animal Care Committee (#852), or by the animal ethics committees of UQAM (no. CIPA 2023–5775). For M-*Bcl6* KO studies, mice were maintained on the C57BL/6 background and were housed under specific pathogen-free conditions with a standard 12:12-h light/dark cycle. Mice had ad libitum access to water and standard irradiated chow (5% fat, Teklad) at the animal facility of Princess Margaret Cancer Research Tower. Whenever possible, age- and sex-matched littermates were used for the studies. For experiments involving intramuscular injections of AAVs, C57BL/6J mice were purchased from Jackson Laboratories. Two to three mice were housed per cage under a 12:12 h light/dark photocycle, with access to a standard chow diet and water available ad libitum.

#### 4.1.2 Genotyping

Genotyping of mice were performed using ear notches of 10-to-14-day-old mice with proteinase K-digestion and PCR (using the Wisent HS- Red Taq Mix) followed by agarose gel electrophoresis on a 2% Sybr safe-stained agarose gel. For primers (Integrative DNA Technologies), see Supplementary Table S1.

### 4.2 Generation of skeletal muscle-specific *Bcl6* knockout (KO) mice

Skeletal-muscle-specific Bcl6 KO mice were generated using the Cre-Lox-P system under control of the *Myf6* promoter, which is highly specific for skeletal muscle [25]. Myf6-Cre ± mice (Jackson Laboratories, 010528) were bred with Bcl6^fl/fl^ mice (Jackson Laboratories, 023727) to generate Myf6-Cre ^+^ BCL6^fl/wt^ mice, which were then intercrossed to generate Myf6-Cre ^+^ Bcl6^fl/fl^ mice, herein referred to as M-*Bcl6* KO. Their wildtype littermates, Myf6-Cre ^+^ BCL6^wt/wt^ mice, herein referred to as M-*Bcl6* WT, and were used as controls.

### 4.3 Generation of adeno-associated virus (AAV) mediated BCL6 knockdown (KD) mice

To silence *Bcl6* in adult mice, 30-week-old wildtype C57BL/6J mice (Jackson Laboratories, Saint-Constant, QC) were injected with AAV containing a U6 promoter and a sequence coding shRNA targeting *Bcl6* (Vector Biolabs), herein referred to as AAV*-shBCL6*. AAV-*Bcl6* was injected intramuscularly (IM) (25 μl per site; 1.5 × 10^11^ gc) into the right TA and GAS muscles. Control AAV containing a U6 promoter and a scramble sh-RNA sequence (Vector Biolabs), herein referred to as AAV-scrmb, was injected into the contralateral (left) limb. We have previously used this approach to successfully modulate protein expression in mouse limb muscles [[62], [63], [64], [65]]. All AAV-sh *Bcl6* experiments were approved by the animal ethics committees of UQAM (no. CIPA 2023-5775).

### 4.4 *In vivo* metabolic studies

Glucose tolerance tests (GTTs) were performed in young (∼15-week-old) and aged (∼52-week-old) mice. Young mice were fasted for 6 h prior to the test, whereas aged mice were fasted overnight (∼16 h). Following the fasting period, mice received an intraperitoneal (i.p.) injection of d-glucose (1 g/kg body weight). Insulin tolerance tests (ITTs) were performed on animals fasted for 4 h using insulin lispro (Lilly) 1 U/kg body mass injected i.p. Blood glucose measurements were obtained at 0, 15, 30, 45, 60, and 120 min after injection using tail vein blood and an GE200 blood glucose-monitoring system. The area under the curve (AUC) was calculated by using the trapezoidal approximation method [66].

### 4.5 Grip strength assessment

The grip strength of forelimbs was measured using a grip strength meter with a metal grid (BIOSEB Bio-BS3; EB Instruments) according to the instructions of the manufacturer. Mice were placed on a metal grid and allowed to grab it with their forelimbs. They were then gently pulled horizontally backward until their grasp was released. Each mouse underwent 15 consecutive trials, and the average of the top three peak force values (in grams) was used for analysis. All measurements were performed by an investigator blinded to mouse genotype. Grip strength values were recorded in Newtons (N) by the apparatus and subsequently converted to gram-force by dividing the force value by the standard gravitational constant (9.80665 m/s^2^) and multiplying by 1,000.

### 4.6 Treadmill exercise

Male, age-matched (∼15-week-old) M-*Bcl6* WT or control littermates were exercised on a motorized treadmill (Columbus Instruments Exer 3/6 Treadmill). All exercised mice were first acclimated to the treadmill using 5 days protocol prior to starting experiments, as described by Furrer et al. [67]. Briefly, exercise training occurred 5 days/week for 1 h for a total of 4 weeks. The first training session was performed at a speed of 10 m/min and was subsequently increased by 0.5 m/min each day, resulting in a final speed of 19.5 m/min after 4 weeks. The inclination of the treadmill was kept constant at 5° for all training sessions.

Following the 4 weeks of exercise training, a maximal performance test was performed on both untrained and trained mice cohorts. Untrained mice were acclimatized to the treadmill prior to the test. The maximal exercise test was performed at an inclination of 5° and, after warming up of 5 min at 5 m/min followed by 5 min at 8 m/min, the speed was progressively increased for 2 m/min every 15 min until exhaustion. Exhaustion was defined as spending 5 consecutive seconds in the “fatigue zone”. The “fatigue zone” is defined as the region encompassing the portion of the treadmill belt within approximately 1 body length of the shock grid as well as the grid, itself, despite mechanical prodding. Untrained and trained mice were sacrificed 48 h after the maximal fatigue test and muscle were collected for downstream analyses.

### 4.7 Analysis of muscle contractility

For M-*Bcl6* KO studies, muscle strength was analyzed using a Dynamic Muscle Data Acquisition and Analysis System (Aurora Scientific, Aurora, ON). *In vivo* maximal tetanic tension was measured as previously described [68]. 12- to 15-week-old mice were anesthetized and positioned on a platform of the muscle testing apparatus (Aurora Scientific Inc.). Hair was removed on the lower right limb, and a needle was inserted through the right knee between two vertical stabilizing posts. Two 28G × 0.5-inch needle probe electrodes were then inserted directly adjacent to the GAS. The right paw was placed on the pedal of a computer-controlled servomotor (301C, Aurora Scientific Inc.). The electrical stimulation was generated from a Grass Stimulator (S88, Grass Technologies, R.I., USA) with connection to the computer (604A, Aurora Scientific Inc). The optimal stimulation was recorded in the computer Dynamic Muscle Control and Analysis Software suite (DMC/DMA, Aurora Scientific Inc).

For AAV-*BCL6* KD experiments, *in vivo* isometric torque of each hindlimb plantar flexor muscles (gastrocnemius, soleus, and plantaris) was assessed in anesthetized mice using a dual-mode servomotor system (1300A: 3-in-1 Whole Animal System–Mouse, Aurora Scientific). Mice were anesthetized with ∼2% isoflurane-mixed with oxygen and placed on a heated platform maintained at 37 °C to preserve body temperature. Hair on the left hindlimb was removed using an electrical clipper. The knee was immobilized using a clamp to ensure isolated ankle joint movement during contractions, while the foot was positioned at a 90° ankle angle and secured to the footplate of the servomotor. The mouse tail was taped loosely to the platform to keep it clear of the foot pedal. The optimal muscle length (Lo) was determined by adjusting the distance between the footplate and the knee while delivering single twitch stimulations (100 mA current, 0.2 ms pulse width) to identify the position yielding maximal twitch force. Once twitch force ceased to increase, this position was used for all subsequent contractions. Stimulation voltage and needle electrode placement were optimized by adjusting the electrical pulse to ensure supramaximal activation. Following optimization, peak isometric torque was measured in response to twitch and then force-frequency relationships were determined at muscle Lo (1, 10, 30, 50, 70. 100, 120 and 150 HZ tetanic stimulation for 500 ms, with 1 min intervals between stimulations to avoid fatigue). The data obtained were analyzed using 611 A Dynamic Muscle Analysis (DMA) software. Torque is determined by the force (mN) applied to, and multiplied by the length of, the foot pedal (m).

### 4.8 Histology and immunohistochemistry of muscle

Muscle samples were mounted in tragacanth on plastic blocks and frozen in liquid nitrogen and stored at −80 °C. Samples were cut into 10 μm cross-sections using a cryostat at −20 °C then mounted on Superfrost slides. The following histological stains were performed on muscle cross-sections: hematoxylin and eosin (H&E) and succinate dehydrogenase (SDH). For all immunohistochemistry experiments except myosin heavy chain immunolabelling, muscle cryosections were first allowed to reach room temperature and rehydrated with phosphate-buffered saline (PBS) (pH 7.2) and then fixed in 4% PFA, permeabilized in 0.1% Triton X-100 in PBS for 15 min. Slides were then washed three additional times with PBS and then blocked with goat serum (10% in PBS) for 1 h. After, muscle sections were incubated with the appropriate primary antibody diluted in 10% goat serum in PBS for 1 h. Sections were then washed three times in PBS before being incubated for 1 h at room temperature with the appropriate secondary antibodies diluted in 10% goat serum in PBS. After washing, stained sections were mounted with Prolong™ Gold with or without DAPI. All primary and secondary antibodies are listed in Supplementary Table S2.

To access muscle fiber type composition, TA muscle sections were immunolabeled for myosin heavy chain (MHC) type I, IIA, and IIB. Muscle sections were washed with phosphate-buffered saline (PBS) (pH 7.2) and blocked with goat serum (10% PBS) for 1h at room temperature. A primary antibody cocktail was added to muscle sections and incubated for 1h at room temperature. The following primary antibody cocktail was used: a mouse IgG2b monoclonal anti-MHC type I, mouse IgG1 monoclonal anti-MHC type IIA, mouse IgM monoclonal anti-MHC type IIB, and a rabbit IgG polyclonal anti-laminin. Muscle sections were then washed three times with PBS and then incubated for 1h at room temperature with the secondary antibody cocktail: Alexa Fluor 350 IgG2b (y2b) goat anti-mouse antibody, Alexa Fluor 594 IgG1 (y1) goat anti-mouse, Alexa Fluor 488 IgM goat anti-mouse, and Alexa Fluor 488 IgG goat anti-rabbit. Muscle sections were then washed in PBS and mounted with Prolong Diamond (Thermo Fisher Scientific). Slides were imaged using an Olympus IX83 fluorescence microscope. Fiber type analyses were performed blinded for experimental group using ImageJ.

### 4.9 *In situ* assessment of mitochondrial respiration in M-*Bcl6* KO and WT mice

Mitochondrial respiration was assessed in permeabilized myofiber bundles with high resolution respirometry, using protocols adapted from previous methods [69,70]. The GAS muscle was excised from anesthetized mice and placed immediately into ice-cold BIOPS buffer containing (in mM) 50 MES Hydrate, 7.23 K_2_EGTA, 2.77 CaK_2_EGTA, 20 imidazole, 0.5 dithiothreitol, 20 taurine, 5.77 ATP, 15 PCr, and 6.56 MgCl_2_·6H_2_O (pH 7.2). Muscles were separated along the longitudinal axis under a dissection microscope into small bundles (∼1–6 mg wet weight) and permeabilized with 40 μg saponin (Sigma Aldrich) in BIOPS on a platform rotor for 30 min at 4 °C, then washed for 15 min at 4 °C in buffer Z containing (in mM) 105 K-MES, 30 KCl, 10 KH_2_PO_4_, 5 MgCl_2_·6H_2_O, 1 EGTA, 5 mg/ml BSA. All bioenergetic assays were performed within 4 h of washing to maintain fiber viability.

Assessments were performed in 2 mL of buffer Z supplemented with 20 mM creatine (creatine-dependent) to saturate mitochondrial creatine kinase activity [71,72]. O_2_ consumption was measured using the Oroboros Oxygraph-2K (Oroboros Instruments, Corp., Innsbruck, Austria) while stirring at 37 °C in the presence of 5 μM blebbistatin to prevent muscle fiber contraction by rigor in response to ADP [70]. Each chamber was oxygenated with 100% pure O_2_ to an initial concentration of 350 μM and experiments were completed before chamber [O_2_] reached 150 μM^70,71^. Before permeabilization, fiber bundles were gently and quickly blotted dry and weighed in 1.5 mL of tared cold BIOPS to ensure fibers remained relaxed. Complex-I supported respiration was stimulated using 5 mM pyruvate and 2 mM malate (State II respiration) followed by titration of ADP concentrations (State III) from physiological ranges (25–100 μM) to supraphysiological (500 μM) and saturating to stimulate maximal coupled respiration (5,000 μM). 10 mM succinate was then supplied to support complex-II respiration.

### 4.10 *In situ* assessment of mitochondrial respiration in mice injected with AAV-scmb and AAV-shBCL6

Mitochondrial function was determined in freshly excised GAS muscles as previously described [64,65]. The muscles were dissected and rapidly immersed in ice-cold stabilizing buffer A (2.77 mM CaK2 ethylene glycol-bis-(2-aminoethylether)-N,N,N,N-tetra-acetic acid (EGTA), 7.23 mM K2 EGTA, 6.56 mM MgCl2, 0.5 mM dithiothreitol (DTT), 50 mM 2-(N-morpholino) ethane-sulfonic acid potassium salt (K-MES), 20 mM imidazole, 20 mM taurine, 5.3 mM Na2 ATP, and 15 mM phosphocreatine, pH 7.3) at 4 °C. The muscles were weighed and then separated into small fiber bundles using fine forceps under a surgical dissecting microscope (Leica S4 E, Germany). The muscle fiber bundles were incubated into a glass scintillation vial for 30 min at low rocking speed containing buffer A supplemented with 0.05 mg/mL saponin to selectively permeabilize the sarcolemma. Fiber bundles were then washed 3 times 10 min at low rocking speed in buffer MiroO5 (0.5 mM EGTA, 6 mM MgCl2, 3 mM H20, 60 mM K-lactobionate, 20 mM Taurine, 10 mM KH2PO4, 20 mM HEPES, 110 mM Sucrose, 1 g/L fatty acid free BSA, pH 7.4 at 4 °C).

The assessment of mitochondrial respiration in permeabilized GAS myofiber was performed in an Oroboros O2K high-resolution fluororespirometer (Oroboros Instruments) at 37 °C in 2 mL of MiRo 5 buffer as previously described [64,65]. Briefly, 3–6 mg (wet weight) of GAS permeabilized fiber bundles were weighed and added to the respiration chamber. The following substrates were added sequentially: 10 mM glutamate plus 5 mM malate (G + M). All respiration experiments were analyzed with MitoFun (https://zenodo.org/records/7510439), an in-house-derived code designed to analyze mitochondrial function data in the Igor Pro 8 software (Wavemetrics, OR, USA) [64].

### 4.11 Transmission electron microscopy (TEM) preparation and analysis

Small strips of tissue were prepared from white GAS fibers, fixed in 2% glutaraldehyde buffer solution in 0.1M cacodylate, pH 7.4, then post-fixed in 1% osmium tetroxide in 0.1M cacodylate buffer. Tissues were dehydrated using a gradient of increasing concentrations of methanol to propylene oxide and infiltrated and embedded in EPONTM at the Facility for Electron Microscopy Research at McGill University. Ultrathin longitudinal sections (60 nm) were cut with a Reichert-Jung Ultracut III ultramicrotome (Leica Microsystems), mounted on nickel carbon-formvar coated grids, and stained with uranyl acetate and lead citrate. Sections were imaged using a Philips EM208S transmission electron microscope operated at 80 kV at the I2E3 platform at Université du Québec à Trois-Rivières. Mitochondrial quantity and morphology were analyzed blinded to genotype using Fiji Image J software, as previously described [35]. Electron microscopy images were quantified for glycogen deposits, characterized by small, dark granules, from 1 (none) to 5 (extremely high), as described before [73]. Representative images for each score were selected and were provided to raters as reference. Scores from six independent, blinded raters were averaged for analysis.

### 4.12 Immunoblotting

Frozen skeletal muscle tissues (∼15 mg) were homogenized using a Bertin Technologies Minilys Personal Homogenizer in ice-cold RIPA buffer (ThermoFisher) supplemented with protease inhibitors (Roche). Proteins in equal quantities (10–20 μg) were loaded onto SDS-PAGE gradient gels (4%–15%, Mini PROTEAN TGX Stain-Free TM Gels, Bio-Rad). Following separation, gels were transferred onto a 0.2 uM PVDF membrane (Bio-Rad) and blocked with 5% milk in TBS-T for 1 h at room temperature. Membranes were then incubated overnight at 4 °C with the commercially available monoclonal rodent OXPHOS cocktail (ab110413; Abcam, Cambridge, UK, 1:500 dilution) including V-ATP5A (55 kDa), III-UQCRC2 (48 kDa), IVMTCO1(40 kDa), II-SDHB (30 kDa), and I-NDUFB8 (20 kDa). Following, membranes were washed (3 × 5 min each time) in TBS-T and incubated at room temperature with appropriate fluorescent secondary antibody. Before detection, membranes were washed in TBS-T (3 × 5 min and then imaged using ChemiDoc imaging system (Bio-Rad) and quantified by densitometry (Fiji ImageJ, http://imagej.nih.gov/ij/). All images were normalized total protein from the stain-free gel. The complete list of antibodies used for immunoblots analyses can be found in Supplementary Table S3. All uncropped western blot images used in the present manuscript are available in Supplementary Fig. 6.

### 4.13 Biochemical analysis of muscle

Gastrocnemius muscle triglycerides were measured in frozen muscle homogenates using a triglyceride colorimetric assay kit (Cat. #10010303, Cayman Chemical). The assay was performed with duplicate samples according to the manufacturer's instructions.

### 4.14 Quantitative real-time PCR

Total RNA from mouse skeletal muscle and non-muscle tissue was isolated by homogenizing snap-frozen tissues in TRIzol Reagent (Invitrogen) and incubating in chloroform followed by precipitation with isopropanol. RNA was quantified using a Nanodrop spectrophotometer and a final concentration of 1uL/10ug was used for cDNA synthesis. mRNA was reverse transcribed with random primers (Invitrogen) using the M-MLV reverse transcriptase enzyme (Invitrogen). Real-time PCR was performed using specific primers and Luna® Universal qPCR Master Mix, on a QuantStudio 7 Flex Real-Time PCR System (Thermo Fisher) and was analyzed using Quantstudio Real-Time PCR Software. The relative mRNA expression was determined by delta–delta CT (ΔΔCt) method after normalizing to the housekeeping genes β-actin, *36b4,* or *18s*. Primer sequences are listed in the Supplementary Information (Table S4).

### 4.15 Mitochondrial DNA (mtDNA) analysis

Purified total DNA was extracted from muscle tissue using the QIAamp DSP DNA Mini Kit (Qiagen) according to the manufacturer's protocol. Samples were then diluted using double-distilled water to obtain a final concentration of 10 ng/uL to be used for real-time PCR. The mtDNA/nDNA ratio was calculated by measuring the expression levels of a mitochondrial-encoded gene (*Cox2* or *16S rRNA*) and normalized to the expression levels of a nuclear-encoded gene (*cyclophilin A: Ppia* or *Hexokinase 2*) following the delta–delta CT method and as previously described [74].

### 4.16 Muscle single-nuclei isolation

TA muscles were isolated from mice immediately following euthanasia. Muscle was placed into lysis buffer and minced with a chilled razor on dry ice. The minced tissue was then further homogenized using glass douncers. Nuclei quality was checked under microscope using a disposable hemocytometer. The nuclei were pelleted by centrifugation for 10 min at 800×*g* at 4 °C and then resuspended in wash buffer for a total of three cycles. Samples were then filtered with a 40 μm cell strainer and then stained for 7-amino-actinomycin D (7-AAD). Nuclei were sorted by flow cytometry to exclude debris and nuclei aggregates by gating 7-AAD positive cells. These collected nuclei were resuspended in wash buffer and pelleted by centrifugation for 10 min at 800×*g* at 4 °C and then resuspended in cold wash and counted.

### 4.17 Construction of gene expression library

snRNA sequencing libraries were generated using the 10X Genomics Chromium Next GEM Single Cell 3’ Reagents Kit (v3.1 Dual index) as per the user guide. Briefly, a Chromium Next GEM chip and reagent master mix were prepared, and nuclei suspensions were loaded onto the chip and partitioned in the Chromium Controller to form Gel Beads-in-Emulsion (GEMs). Reverse transcription and cDNA cleanup were performed according to the kit instructions. cDNA was amplified by PCR (cycles per 10x guidance), and samples were run on a Bioanalyzer (Agilent Technologies) to determine cDNA concentration. 10x barcoded sequencing library was prepared and subjected to Illumina sequencing. Alignment, filtering, quantification, feature-barcode matrices generation and demultiplexing was performed using the 10x Genomics Cell Ranger pipeline retaining 50, 000 reads per nuclei.

### 4.18 Single nuclei RNA sequencing analysis

Single nuclei RNA sequencing data was analyzed using Seurat (version 5.0.3) [75] in R package (version 4.2.1) (https://www.R-project.org/). Low quality cells expressing less than 200 genes and low expressed genes present in less than 3 cells were removed. Cells with high mitochondrial DNA, high number of unique feature counts (potential doublets or multiplets) were excluded. We used DoubletFinder (version 2.04) [76] for the detection of doublets. Samples were merged for subsequent clustering and visualization. The batch correction between samples was done with the Harmony R package (version 1.0) [77]. Data was normalized using the log normalization method and highly variable genes were detected using variance-stabilizing transformation (vst). The normalized data was centered and scaled. Dimensionality reduction was performed using principal component analysis (PCA) and the most significant PCs were chosen for subsequent clustering. Graph-based clustering was implemented by calculating k-nearest neighbors, followed by modularity optimization to clusters cells. Non-linear dimensionality reduction and visualization was performed using UMAP (Uniform Manifold Approximation and Projection). Clusters were annotated on the basis of canonical markers and differential gene expression testing was used to determine a gene set signature for each cluster using the Wilcoxon Rank Sum test. GO enrichment analysis was performed on differentially expressed genes using enrichGO as implemented in clusterProfiler package (v 4.4.4) [78].

### 4.19 Statistical analysis

The data is presented as ± S.E.M. Unless indicated otherwise, the data was analyzed using two-tailed Student's unpaired *t* test for comparisons between two independent groups. Comparisons between more than two groups were performed using either a one-way or two-way Analysis of Variance (ANOVA). Unless otherwise indicated, corrections for multiple comparisons were performed using the two-stage step-up Benjamini, Krieger, and Yekutieli method (*p* < 0.05 and *q* < 0.1 were considered significant). All statistical analyses were performed using GraphPad Prism 10.3.1.

## CRediT authorship contribution statement

**Shirine E. Usmani:** Writing – review & editing, Writing – original draft, Visualization, Validation, Methodology, Investigation, Funding acquisition, Formal analysis, Data curation, Conceptualization. **Jean-Philippe Leduc-Gaudet:** Writing – review & editing, Writing – original draft, Visualization, Validation, Supervision, Methodology, Investigation, Formal analysis, Data curation, Conceptualization. **Stephanie Chau:** Writing – review & editing, Writing – original draft, Visualization, Investigation, Formal analysis, Data curation. **Catherine A. Bellissimo:** Writing – review & editing, Validation, Methodology, Formal analysis, Data curation. **Shabana Vohra:** Writing – review & editing, Visualization, Validation, Methodology, Investigation, Formal analysis, Data curation. **Krystel Desjardins:** Writing – review & editing, Visualization, Formal analysis, Data curation. **Evan Pollock-Tahiri:** Writing – review & editing, Validation, Formal analysis, Data curation. **Irisa Shi:** Writing – review & editing, Formal analysis, Data curation. **Felice Chun:** Writing – review & editing, Formal analysis, Data curation. **Anthony Capobianco:** Writing – review & editing, Visualization, Formal analysis, Data curation. **Pascale Delisle:** Writing – review & editing, Visualization, Formal analysis, Data curation. **Wanda Dupebe:** Writing – review & editing, Visualization, Formal analysis, Data curation. **Marina Cefis:** Writing – review & editing, Formal analysis, Data curation. **Vincent Marcangeli:** Writing – review & editing, Formal analysis, Data curation. **Martha Ghebreselassie:** Writing – review & editing, Formal analysis, Data curation. **Yu-Cheng Liang:** Data curation, Formal analysis, Investigation, Writing – review & editing. **Yasufumi Seki:** Data curation, Formal analysis, Investigation, Writing – review & editing. **Dominique Mayaki:** Writing – review & editing, Formal analysis, Data curation. **Sabah Hussain:** Writing – review & editing, Validation, Supervision, Methodology, Funding acquisition, Data curation. **Ewan Goligher:** Writing – review & editing, Formal analysis, Data curation. **Mohit Kapoor:** Writing – review & editing, Validation, Supervision, Methodology, Funding acquisition, Formal analysis, Data curation. **Marius Locke:** Writing – review & editing, Supervision, Methodology, Formal analysis, Data curation. **Gilles Gouspillou:** Writing – review & editing, Writing – original draft, Visualization, Validation, Supervision, Software, Resources, Project administration, Methodology, Investigation, Funding acquisition, Formal analysis, Data curation, Conceptualization. **Minna Woo:** Writing – review & editing, Writing – original draft, Visualization, Validation, Supervision, Software, Resources, Project administration, Methodology, Investigation, Funding acquisition, Formal analysis, Data curation, Conceptualization.

## Funding sources

This work was funded by grants from the 10.13039/501100000024Canadian Institutes of Health Research (CIHR) DMT-468314 to MW, 10.13039/100024172SH, MK, GG, and SU, and 10.13039/501100000024CIHR PJT 159505 to MW. MW is supported by the Ajmera Chair in Molecular Diabetes Research. SU was supported by the Eliot Phillipson Clinician Scientist Training Program from the Department of Medicine and postdoctoral fellowship from the 10.13039/501100000064Banting and Best Diabetes Centre at the University of Toronto. GG is supported by a Chercheur Boursier Senior salary award from the 10.13039/501100000156Fonds de Recherche du Québec en Santé (FRQS-365892; https://doi.org/10.69777/365892) and a 10.13039/501100000038Natural Sciences and Engineering Research Council of Canada Discovery Grant (NSERC-DG: RGPIN-2021-03724). JPLG is supported by NSERC-DG (RGPIN-5033-2024) and by the Banting Discovery Foundation. AC was supported by undergraduate scholarships from the 10.13039/501100000038NSERC (599099). VM is supported by a Ph.D. stipend from the 10.13039/501100000156FRQS. MC was supported by a postdoctoral fellowship from the FRQS. SC is supported by the Canadian Graduate Scholarship-M from CIHR. CAB is supported by a postdoctoral fellowship from CIHR. The funders had no role in the design of the study, in the collection, analyses, or interpretation of data; in the writing of the manuscript, or in the decision to publish the results.

## Declaration of competing interest

The authors declare that they have no known competing financial interests or personal relationships that could have appeared to influence the work reported in this paper.

## Data Availability

The GEO accession number of our snRNAseq data should be listed here (GSE306646). Readers may want to access these data.
