# Supplementary material for: BCL6 regulates skeletal muscle mass and mitochondrial bioenergetics
Source: Mol Metab. 2026 Apr 13;108:102367. doi: 10.1016/j.molmet.2026.102367 (PMC13141646; doi:10.1016/j.molmet.2026.102367)
Supplement: Multimedia component 1 [file mmc1.docx]

**Supplementary Tables**

**Table S1: List of primers for genotyping**

| **Gene** | **Sequence** |
| --- | --- |
| *Myf6 -Cre* | WT-1: 5’ CTTCAGCGCCTTTCTTCCATCG 3’  WT-2: 5’ GAACTTCGTTTGCAACTGAC 3’  MUT: 5’ GGATAGTGAAACAGGGCAA 3’ |
| *Bcl6* | F : GTGTCCTGGGGTTACAGGTG  R : CCTGTCCTGCCTACCCATAG |

# Table S2: Antibodies used for immunofluorescence studies.

| **Antibody** | **Source / Product no.** | **Dilution** | **Analysis** |
| --- | --- | --- | --- |
| rabbit IgG polyclonal anti-laminin | Sigma-Aldrich # L9393 | 1 :750 | IF |
| mouse IgG2b monoclonal anti-MHC type I | DSHB # BA-F8 | 1:25 | IF |
| mouse IgG1 monoclonal anti-MHC type IIa | DSHB # SC-71 | 1 :200 | IF |
| mouse IgM monoclonal anti-MHC type IIb | DSHB # BF-F3 | 1 :200 | IF |
| Alexa Fluor 350 IgG2b (y2b) goat anti-mouse | Invitrogen, A-21140 | 1:500 | IF |
| Alexa Fluor 488 IgG goat anti-rabbit | Invitrogen, A-11008 | 1:500 | IF |
| Alexa Fluor® 488 IgG2a goat anti-mouse | ThermoFisher, A-21131 | 1:500 | IF |
| Alexa Fluor 594 IgG1 (y1) goat anti-mouse | Invitrogen, A-21125 | 1:100 | IF |
| Alexa Fluor 594 IgG goat anti-rabbit | Invitrogen, A-11037, | 1∶500 | IF |
| Alexa Fluor IgG goat anti-rabbit | ThermoFisher, A-11011 | 1:500 | IF |

# *All primary antibodies targeting MHCs were purchased from the Developmental Studies Hybridoma Bank (DSHB, University of Iowa, IA). Table S3: Antibodies for immunoblotting

| **Antibody** | **Source / Product no.** | **Dilution** | **Analysis** |
| --- | --- | --- | --- |
| OXPHOS | Abcam #110413 | 1/500 | WB |
| OPA1 | Cell Signalling Technology # 80471 | 1/1000 | WB |
| DRP1 | Cell Signalling Technology # 14647 | 1/1000 | WB |
| VDAC | Cell Signalling Technology # 4661 | 1/1000 | WB |
| MFN2 | Cell Signalling Technology # 9482 | 1/1000 | WB |
| Retreg1 (FAM134B) | Cell Signalling Technology # 83414 | 1/1000 | WB |
| Akt | Cell Signalling Technology #9272 | 1/1000 | WB |
| Phospho-Akt (Ser473) | Cell Signalling Technology #9271 | 1/1000 | WB |
| GAPDH | Cell Signalling Technology #92218 | 1/1000 | WB |
| Goat anti rabbit IgG | Cell Signalling Technology # 7074 | 1/5000 | WB |
| Anti-mouse IgG | Cell Signalling Technology # 7076 | 1/5000 | WB |

**Table S4: List of primers for RT-qPCR**

|  | **Forward primer (5’-3’)** | **Reverse primer (5’-3’)** |
| --- | --- | --- |
| *Bcl6* | GCCACAAGACTGTCCACACG | CTCTCCAGTGTGGATGAGCAC |
| *Mfn1* | GCTGTCAGAGCCCATCTTTC | CAGCCCACTGTTTTCCAAAT |
| *Mfn2* | ATGTTACCACGGAGCTGGAC | AACTGCTTCTCCGTCTGCAT |
| *Opa1* | ATACTGGGATCTGCTGTTGG | AAGTCAGGCACAATCCACTT |
| *Drp1* | TCAGATCGTCGTAGTGGGAA | TCTTCTGGTGAAACGTGGAC |
| *Fis1* | AAGTATGTGCGAGGGCTGT | TGCCTACCAGTCCATCTTTC |
| *Parkin* | CCTCTGTCATCTGGTGCCT | GCTAAGCGGTAAAGAAATCAA |
| *Pink1* | TCTCAAGTCCGACAACATCCT | TTGCCACCACGCTCTACAC |
| *Nix* | TCTTCCTTTCTCATGTGCTGG | GCTTTTCGTCTCCCTCAGTAG |
| *B-actin* | CATTGCTGACAGGATGCAGAAGG | TGCTGGAAGGTGGACAGTGAGG |
| *Socs2* | GCGCGTCTGGCGAAAGCCCT | GAAAGTTCCTTCTGGAGCCTCTT |
| *Igf-1* | GTGGATGCTCTTCAGTTCGTGTG | TCCAGTCTCCTCAGATCACAGC |
| *Tfam* | GCACCCTGCAGAGTGTTCAA | CGCCCAGGCCTCTACCTT |
| *Tfb1m* | TGCGTTTCAGTTTCGAAGGA | TCGAGGCGTTGTGCTTCAG |
| *Tfb2m* | TTTCCACTTGGTAAAGCATTGCT | ATCAACCGTACTCAGTGAACGTAA |
| *Cox2* | CCATAGGGCACCAATGATACTG | AGTCGGCCTGGGATGGCATC |
| *16S* | CCGCAAGGGAAAGATGAAAGAC | TCGTTTGGTTTCGGGGTTTC |
| *Cyclophilin:A Ppia* | ACACGCCATAATGGCACTGG | CAGTCTTGGCAGTGCAGAT |
| *Hexokinase 2* | GCCAGCCTCTCCTGATTTTAGTGT | GGGAACACAAAAGACCTCTTCTGG |
| *36b4* | AGATGCAGCAGATCCGCA | GTTCTTGCCCATCAGCACC |
| *Meg3* | CGGCTCACACCAGTCTTCCA | AGCAGGTACTCGAGCAGCAG |
| *18s* | GCGCTCATCTTTGCCGTAGT | CGATCCGAGGGCCTCACTA |

**Supplementary Figures**

**Figure S1: *Bcl6* does not alter glucose homeostasis.** (A) Body mass difference of M-*Bcl6* WT and M-*Bcl6* KO female mice at the indicated times. *n* = 5-13 per group. (B) Body mass of male M-*Bcl6* WT and M-*Bcl6* KO mice at 1 year of age. *n* = 11-17 per group. (C) Body mass of female M-*Bcl6* WT and M-*Bcl6* KO mice at 1 year of age. *n* = 12-16 per group. (D) Glucose tolerance test (GTT) (left) and area under the curve (AUC) (right) and (E) insulin tolerance test (ITT) and AUC in male M-*Bcl6* WT and M-*Bcl6* KO mice at 15-18 weeks of age. *n* = 4-5 per group. (F) GTT (left) and AUC (right) and (G) ITT (left) and AUC (right) in female M-*Bcl6* WT and M-*Bcl6* KO mice at 15-18 weeks of age. *n* = 4-6 per group. (H) GTT (left) and AUC (right) and (I) ITT (left) and AUC (right) in female M-*Bcl6* WT and M-*Bcl6* KO mice at 1 year of age. *n* = 5-10 per group. (J) GTT (left) and AUC (right) and (K) ITT (left) and AUC (right) in male M-*Bcl6* WT and M-*Bcl6* KO mice at 1 year of age. *n* = 5-13 per group. Data from A, D-K were analyzed with two-way ANOVA, and corrections for multiple comparisons were performed with the two-stage step-up method of Benjamini, Krieger, and Yekutieli (∗p < 0.05 and q < 0.1). Data in B and C were analyzed with paired two-tailed Student’s *t*-test (*p < 0.05 and **p < 0.01). Data are presented as mean ± SEM.

**Figure S2: *Bcl6* regulates muscle mass and strength.** (A) Tissue mass of various muscle groups from female M-*Bcl6* WT and M-*Bcl6* KO mice at 10-12 weeks of age and (B) normalized to body mass. *n* = 7-15 per group. (C) Tissue mass of liver, subcutaneous white adipose tissue (sWAT), epididymal white adipose tissue (eWAT), brown adipose tissue (BAT), spleen, pancreas, heart, and lung from male M-*Bcl6* WT (*n =* 8) and M-*Bcl6* KO (*n* = 8) mice and (D) female M-*Bcl6* WT (*n =* 15) and M-*Bcl6* KO (*n* = 8) mice. (E) Tissue mass of various muscle groups from male M-*Bcl6* WT and M-*Bcl6* KO mice at 1 year of age. Normalized data are shown as percent of M-*Bcl6* WT. *n* = 6 per group. (F) Tissue mass of various muscle groups from female M-*Bcl6* WT and M-*Bcl6* KO mice at 1 year of age. Normalized data are shown as percent of M-*Bcl6* WT. *n* = 7-9 per group. (G) Absolute forelimb grip strength measured in female M-*Bcl6* WT and M-*Bcl6* KO mice at 8 weeks of age. *n* = 15-21 per group. (H) Forelimb grip strength normalized per body mass measured in male M-*Bcl6* WT (*n =* 13) and M-*Bcl6* KO (*n* = 14) mice and (I) female M-*Bcl6* WT (*n =* 15) and M-*Bcl6* KO (*n* = 8) mice at 8 weeks of age. (J) Absolute forelimb grip strength (left) and normalized per body mass (right) measured in male M-*Bcl6* WT and M-*Bcl6* KO mice at 1 year of age. *n* = 8-12 per group. (K) Absolute forelimb grip strength (left) and normalized per body mass (right) measured in female M-*Bcl6* WT and M-*Bcl6* KO mice at 1 year of age. *n* = 10-15 per group. Data in A, B, C, D, E and F were analyzed with two-way ANOVA, and corrections for multiple comparisons were performed with the two-stage step-up method of Benjamini, Krieger, and Yekutieli (∗p < 0.05 and q < 0.1). Comparisons in G-K were performed using unpaired two-tailed Student’s *t*-tests (*p < 0.05 and **p < 0.01). Data are presented as mean ± SEM (with individual data points).

**Figure S3: Top upregulated and downregulated biological processes in M-*Bcl6* KO mice.** Top upregulated (left) and downregulated (right) biological processes in M-*Bcl6* KO mice obtained using snRNA-seq data in the (A) Type IIb-1 myonuclear cluster, (B) Type IIb-2 myonuclear cluster, (C) Type IIa/IIx-1 myonuclear cluster and (D) Type IIa/IIx-2 myonuclear cluster.

**Figure S4: Loss of *Bcl6* increases the expression of lncRNA in the Dlk1-Dio3 locus.** (A) UMAP plot of the myonuclear gene expression of *Meg3* in 6-week-old male M-*Bcl6* WT and M-*Bcl6* KO mice derived from snRNA-seq data. (B) Quantification of mRNA expression of *Meg3* in various muscle of 8-week-old male M-*Bcl6* WT and M-*Bcl6* KO mice assessed by RT-qPCR. *n =* 4-8 per group. (C) Quantification of mRNA expression of *Meg3* in quadricep muscle of 4-week-old female M-*Bcl6* WT and M-*Bcl6* KO mice assessed by RT-qPCR. *n* = 3-4 per group. (D) snATAC-seq data showing increased chromatin accessibility for *Meg3* in 6-week-old male M-*Bcl6* WT vs M-*Bcl6* KO mice*.* (E) UMAP plot of the myonuclear gene expression of *Rian* in 6-week-old male M-*Bcl6* WT and M-*Bcl6* KO mice derived from snRNA-seq data. (F) UMAP plot of the myonuclear gene expression of *Mirg* in 6-week-old male M-*Bcl6* WT and M-*Bcl6* KO mice derived from snRNA-seq data.

** Figure S5: Loss of *Bcl6* increases glycogen content but does not affect intramyocellular lipid levels**. (A) Muscle glycogen quantification using a predefined grading system (1-5), analyzed from electron micrographs of M-*Bcl6* WT and KO GAS muscle. (B) Triglyceride content in the gastrocnemius (GAS) muscle in 20-week-old male M-*Bcl6* WT and M-*Bcl6* KO mice. *n* = 4-5 per group. Comparisons in A and B were performed using unpaired two-tailed Student’s *t*-tests (*p < 0.05 and **p < 0.01). Data are presented as mean ± SEM (with individual data points).

**Figure S6: Uncropped western blot images.**
